# Efficacy versus effectiveness of clinical genetic testing criteria for BRCA1 and BRCA2 hereditary mutations in incident breast cancer

**DOI:** 10.1007/s10689-016-9953-x

**Published:** 2017-01-24

**Authors:** Martin P. Nilsson, Christof Winter, Ulf Kristoffersson, Martin Rehn, Christer Larsson, Lao H. Saal, Niklas Loman

**Affiliations:** 1 Division of Oncology and Pathology, Department of Clinical Sciences, Lund University, Lund, Sweden; 2 Department of Haematology, Oncology and Radiation Physics, Skåne University Hospital, Lund, Sweden; 3 Institute of Clinical Chemistry and Pathobiochemistry, Klinikum rechts der Isar, Technische Universität München, Munich, Germany; 4 Department of Clinical Genetics, Laboratory Medicine Region Skåne, Lund, Sweden; 5 Department of Clinical Genetics, Lund University, Lund, Sweden; 6 Department of Surgery, Skåne University Hospital, Malmö, Sweden; 7 Department of Translational Cancer Research, Lund University, Lund, Sweden; 8 CREATE Health Strategic Centre for Translational Cancer Research, Lund University, Lund, Sweden

**Keywords:** BRCA1, BRCA2, Genetic testing, Testing criteria, Effectiveness, Breast cancer

## Abstract

Increasing evidence supports the benefit of identifying *BRCA1* and *BRCA2* germline mutations in early breast cancer. Selection of patients for genetic testing is based on defined criteria taking individual and family history related factors into account. It is important to make a distinction between *efficacy* and *effectiveness* of BRCA testing criteria. *Efficacy* can be defined as the performance under ideal circumstances, whereas *effectiveness* refers to its real life performance. To allow for an unbiased and detailed evaluation of efficacy and effectiveness of the Swedish BRCA testing criteria, we retrospectively analyzed a prospectively collected cohort of 273 breast cancer patients from the well-characterized, population-based, single-site All Breast Cancer in Malmö (ABiM) study. The patients were diagnosed with breast cancer during the years 2007 through 2009. Out of 20 mutation carriers identified, 13 fulfilled Swedish criteria at time of diagnosis. Thus, the efficacy of these criteria was 65%. Excluding three patients in whom a mutation was already known at time of diagnosis, only 3/17 had been identified in the clinical routine, corresponding to an effectiveness of 18%. Here we detail the reasons why mutation carriers in our cohort were not detected though routine health care. In conclusion, effectiveness of BRCA testing criteria was much lower than efficacy. Our results indicate that current testing criteria and procedures associated with *BRCA1* and *BRCA2* testing are insufficient. There is room for improvement of their efficacy, but even more so regarding effectiveness. Clinical BRCA testing routines need to be critically revised.

**Electronic supplementary material:**

The online version of this article (doi:10.1007/s10689-016-9953-x) contains supplementary material, which is available to authorized users.

## Introduction

*Efficacy* can be defined as the performance of an intervention under ideal and controlled circumstances. *Effectiveness* refers to the performance of an intervention under “real world” circumstances [1]. The distinction between efficacy and effectiveness is important but often poorly understood [2]. In the following, we use the term *efficacy* to denote the proportion of *BRCA1* or *BRCA2* mutation carriers among newly diagnosed breast cancer patients that fulfilled selection criteria of screening for mutations (BRCA testing criteria), and we use the term *effectiveness* to denote the proportion of carriers that were actually tested as probands during their interactions with health care, under real world circumstances.

The benefit of *BRCA1* or *BRCA2* genotyping in newly diagnosed breast cancer patients is increasingly evident. Some studies have demonstrated benefit in terms of an increased survival associated with risk-reducing bilateral salpingo-oophorectomy [3], and—in the long run—also for the risk-reducing removal of the contralateral breast in *BRCA1* and *BRCA2* mutation carriers [4, 5].

Many studies have investigated efficacy of various BRCA testing criteria [6–9]. However, the results of these efficacy studies cannot be generalized to real world circumstances, because a mutation carrier can evade detection for other reasons than not fulfilling the selection criteria: i.e. incomplete family history reported or recorded, was not referred, did not pursue testing etc. In recent years, studies on effectiveness of BRCA testing criteria have also been published [9–22]. However, due to a rather small number of studies from only a few countries, and study designs subjected to bias, the effectiveness of such criteria is still remarkably unclear. Of note, regarding most clinical aspects, effectiveness is a more important measure of diagnostic success than efficacy. When designing BRCA testing procedures the whole process of identifying, ascertaining and sampling of potential patients for BRCA testing, must be taken into consideration.

For valid comparisons with other methods of identifying mutation carriers, such as universal testing of all breast cancer patients, it is important to know and account for the difference between efficacy and effectiveness of current BRCA testing criteria. Furthermore, detailed information about the reasons why effectiveness (adherence to) is lower than efficacy (sensitivity) can help directing interventions aimed at improving effectiveness [10, 14, 16–18, 21, 23–26].

The All Breast Cancer in Malmö (ABiM) study, a well-characterized, population-based, prospectively collected cohort of 273 breast cancer patients, diagnosed during the period of 2007–2009 at a single institution, was retrospectively analyzed for the presence of germline *BRCA1* and *BRCA2* mutations. The study design allows for an unbiased and detailed comparison between efficacy and effectiveness of BRCA testing criteria in a European nation with modern oncogenetic testing criteria and no dominating germline founder mutations. Also, specific reasons why a majority of the mutation carriers were not detected though routine health care are detailed.

## Materials and methods

### Malmö and Sweden: general background

Malmö is a city located in South Sweden. It has only one public hospital (SUS Malmö), which serves a catchment population of approximately 400,000 inhabitants. There are no private hospitals performing breast cancer surgery in the region, and >99% of the breast cancer patients that are residents of the catchment area have primary surgery at SUS Malmö. Healthcare co-payment is limited to a maximum of 1100 SEK (~127 USD) per 12 months, which is reached as a consequence of primary breast cancer surgery. Therefore, any further investigations within 12 months are free of charge for the patient. Furthermore, since genetic counseling and testing for hereditary cancers are covered by a separate budget; referral, counseling and BRCA testing does not incur any cost for the referring clinic or the patient.

In the South Sweden Health Care Region, with approximately 1.5 million inhabitants and including Malmö, genetic counseling and genetic testing for hereditary breast cancer is only performed at the Hereditary Cancer Unit (HCU) of the Department of Clinical Genetics, SUS Lund. The process from referral to testing is the following:

1. A patient is referred to the HCU, usually by a surgeon or by an oncologist. Patients can also self-refer. All referred patients are registered in a searchable database (The Onkgen database).
2. The HCU sends out a family history questionnaire to the patient by mail.
3. When the questionnaire is returned, cancer diagnoses in relatives are confirmed, if possible. Then, the patient is invited to a one-hour face-to-face genetic counseling session at the HCU. If the family fulfills the Swedish Breast Cancer Group criteria for screening of mutations in *BRCA1* and *BRCA2* (Table 1), genetic testing is offered.

**Table 1:** Swedish Breast Cancer Group criteria for screening of mutations in *BRCA1* and *BRCA2*

Three cases of breast cancer in first degree relatives, or second degree relatives thought a male, with at least one diagnosed ≤age 50, and/or ovarian cancer (regardless of age)	
Two cases of breast cancer or ovarian cancer in first degree relatives, or second degree relatives thought a male, with at least one case of breast cancer diagnosed ≤age 40, or two cases of ovarian cancer (regardless of age)	
One case of breast cancer ≤age 35	
One case of triple-negative breast cancer ≤age 40^a^	
One case of male breast cancer	
Breast cancer and ovarian cancer in one individual.	

Cases of bilateral breast cancer, prostate cancer, and pancreatic cancer may strengthen the indication for screening of mutations in *BRCA1* and *BRCA2*, but are not defined in any specific criterion

^a^This criterion was not fully applied during the study period; however this does not affect the conclusions of the study
4. A patient that has not returned the family history questionnaire is sent one reminder by mail after about 6 months. Usually, but not always, the patient is also telephoned by a genetic counselor, to find out why the patient did not return the questionnaire. If the patient does not return the questionnaire after the reminder, an answer of the referral is sent back to the referring clinician.
5. Test results are delivered at a 30–60 min face-to-face session at the HCU.

The Swedish Breast Cancer Group criteria for screening of mutations in *BRCA1* and *BRCA2* (current criteria for 2015 listed in Table 1) are the only BRCA testing criteria used in Sweden. These criteria have a long history, dating back to the mid-nineties when a preceding variant of them was suggested and implemented by the founding members of the Swedish Cancer Society-funded Oncogenetic Planning Group. Clinicians and health care institutions do not have any obligation to follow them, but most surgeons and oncologists are familiar with them. They are easy to interpret, but more exclusory than BRCA testing criteria in many other countries. Since the start of the ABiM study in 2007, the following changes have been adopted to the Swedish criteria: “One case of triple-negative breast cancer ≤age 40”, and “Cases of prostate cancer and pancreatic cancer may strengthen the indication for screening of mutations in *BRCA1* and *BRCA2*, but are not defined in any specific criterion” have been added. Applying the criteria used in 2007 or the criteria used in 2015 to our study population did not change any results in our study. Family history of prostate cancer and pancreatic cancer was not documented in the medical records by the surgeons and the oncologists in 2007–2009, most likely because those cancer diagnoses were not part of the testing criteria at that time. Therefore, prostate cancer and pancreatic cancer in the family has not been taken into account in our study.

To the best of our knowledge, BRCA testing by other routes during the study period has been limited in our region. Some patients have been tested as part of clinical research protocols, aiming at the inclusion into clinical trials, but during the catchment period of the current study this was very limited and no patients in the current data set underwent testing within one of those protocols. Testing based on the initiative from patients themselves, sending samples to commercial test providers is also limited in Sweden, and was probably non-existing during the period 2007–2009.

### Study population

The study population has been described in detail previously [27]. Briefly, patients diagnosed with invasive breast cancer who were scheduled for surgery during the years 2007 through 2009 in Malmö, Sweden, were asked to participate in the population-based All Breast Cancer in Malmö (ABiM) study by agreeing to donate a blood sample for research purposes and to consent the use of blood and tumor tissues for molecular analyses. Approximately 80% of all invasive breast cancer patients in Malmö who were scheduled for surgery during the period of interest were included in the ABiM study (n = 538); the remaining 20% were not asked (due to inability to understand written Swedish, psychological reasons, or other unspecified reasons) or declined to participate. In the study information sheet given to the patients, nothing was written about hereditary breast cancer, and at study entry the patients did not expect to be recontacted at a later point of time. Accordingly, patients were not biased to participate because of a wish to find out whether their breast cancer was of a hereditary type or not. No research tissue was taken unless it was certain not to influence the quality of diagnostic procedures. As a consequence, as well due to the quantity requirements of DNA and the quality requirements of sequencing data, for the present study we analyzed 273 patients; they constitute our study population. Comparisons between the study population and the patients from the ABiM cohort that were not included in the present study population are presented in Supplementary Table S1. Compared to the ABiM patients not analyzed herein, patients included in the study population differed significantly with respect to tumor size, grade, Ki-67, St. Gallen subtype, and adjuvant treatment (Supplementary Table S1).

In 2014, analyses of germline and tumor DNA, stored from time of diagnosis, were conducted within a biobank research study. As previously reported, pathogenic germline mutations in *BRCA1* (n = 10) or *BRCA2* (n = 10) were detected in 20 patients (7%) [27].

For each of these 20 mutation carriers, we identified which cases had or had not been tested within routine health care by reviewing the medical records and cross-referencing the Onkgen database (see *Malmö and Sweden: general background*). Then, in 2015, for the patients that were unaware of their mutation carrier status, we recontacted the patients or, for deceased patients, their next of kin. The families were invited to the HCU where a detailed family history of cancer at the time of diagnosis was obtained.

For the present study, the following information was extracted from the 20 mutation carriers’ surgical and oncological medical records: date of breast cancer diagnosis, documented personal and family history of cancer at time of diagnosis, date of referral to the HCU, and any comments about genetic counseling or genetic testing. The documented family history of cancer in the surgical and oncological medical records was then compared with the information about family history at time of diagnosis in the HCU medical records; for discrepancies, the information in the HCU medical records took precedence.

The ABiM study was conducted in accordance with the Declaration of Helsinki and has been approved by the Regional Ethical Review Board in Lund (diary numbers 2007/155, 2009/10, and 2009/658). Written information was given by trained health professionals and all patients provided written informed consent.

### Definitions

We define a *proband* as the first person in a family that was referred for genetic counseling.

We define *efficacy* as the proportion of mutation carriers among the breast cancer patients in our study population that fulfilled the Swedish BRCA testing criteria at time of breast cancer diagnosis.

We define *effectiveness* as the proportion of mutation carriers among the breast cancer patients in our study population that were referred for genetic counseling as probands, without the occurrence of new incident cases of breast cancer or ovarian cancer in the family, and subsequently actually underwent genetic testing. Patients, in whom a mutation was already known in the patient or in the family at time of breast cancer diagnosis, were excluded from the analysis of effectiveness.

We define *predictive testing* as a genetic analysis of the presence, or absence, of a mutation previously identified in a relative.

## Results

Pathogenic germline mutations in *BRCA1* (n = 10) or *BRCA2* (n = 10) were detected retrospectively in a biobank research setting, 5–7 years after breast cancer diagnosis, in 20 out of 273 breast cancer patients [27]. According to the family history as it was documented by the treating physicians at time of diagnosis, 11 out of 20 mutation carriers fulfilled the Swedish BRCA testing criteria (listed in Table 1). For 3 patients, a BRCA mutation was already known in the patient or in the family at time of diagnosis. Of the remaining 8 patients, 6 were referred to the Hereditary Cancer Unit (HCU) for genetic counseling and genetic testing; 5 of them within 6 months after diagnosis, and one of them 2 years and 8 months after diagnosis. Two of those 6 patients did not attend genetic counseling, and where thus not tested, because they did not return the family history questionnaire to the HCU (see “Materials and methods” section). Of 4 patients attending genetic counseling, 3 patients were tested (all were *BRCA1* mutation carriers). The forth patient was informed at the genetic counseling session that another person in her family should be tested first, since that person was diagnosed with cancer at the youngest age in the family; however, that person lived abroad and to our knowledge was never tested.

In 2015, we recontacted the patients (or next of kins of deceased patients) who were not aware of their carrier status. We were thus retrospectively able to thoroughly assess the family history of cancer. This revealed that another 2 patients actually fulfilled the Swedish BRCA testing criteria at time of diagnosis, although this had not been documented in the medical records of the treating physicians at the time. One of those patients was aware of ovarian cancer at the age of 50 in her mother, but this was not documented (we do not know if it was not asked about, or just not noted by the treating physician). The other patient was aware of a cancer diagnosis in her aunt, but not the type of cancer. Following confirmation of the aunt's diagnosis in the cancer registry, it was confirmed that the aunt had been diagnosed with both breast cancer and ovarian cancer. Table 2 summarizes the reasons for carriers not being tested.

**Table 2:** Reasons for BRCA mutation carriers not tested as probands following breast cancer diagnosis

N	Reason for not being tested as probands
7	Did not fulfill the Swedish BRCA testing criteria
2	Family history not reported/assessed/documented correctly in the medical records, thus appeared as not fulfilling the Swedish BRCA testing criteria
3	BRCA mutation already known in the patient/family
2	Not referred for GC
2	Referred for GC, but did not attend GC
1	Attended GC, but not tested

Out of 20 carriers, 17 were not tested as probands

*GC* Genetic counseling

Taken together, 13 out of 20 mutation carriers in this cohort actually fulfilled the Swedish BRCA testing criteria at time of breast cancer diagnosis (7 *BRCA1* and 6 *BRCA2*). Accordingly, the efficacy of the testing criteria was 65% (13/20). Excluding the three patients/families in whom a mutation was already known at time of diagnosis, 17 mutation carriers remained that could potentially have been identified. We conclude that only 3 of them were identified as probands and tested as a consequence of the cancer genetic counseling process, corresponding to an effectiveness of the testing criteria of 18% (3/17).

Over the 5–7 years that have elapsed since their diagnosis of breast cancer, another 3 patients from our cohort have been identified as mutation carriers as the result of them undergoing predictive testing of a pathogenic BRCA mutation found in a close or distant relative, where the relative was the proband. In none of these cases was the pathogenic mutation identified in the relative at the time of diagnosis of breast cancer in the patient from our cohort. Furthermore, none of these relatives were referred for testing as a consequence of the case of breast cancer in the patient from our cohort; instead, the reason for testing was prevalent and new incident cases of breast cancer and ovarian cancer in relatives. In other words, these 3 patients from our cohort were not identified as mutation carriers as a consequence of their own diagnosis of breast cancer, and therefore they do not contribute to the effectiveness ratio in our study, according to our definition of effectiveness (see “Materials and methods” section for definition).

## Discussion

Germline BRCA mutations are already today a treatment predictive factor for the selection of patients for risk reducing surgeries, where an increasing body of evidence underscores the benefit in terms of survival in cases of early breast cancer; the importance is expected to increase further with the advent of directed medical interventions for BRCA-mutation carries with cancer. Thus, the process of identifying germline BRCA mutation status in early breast cancer is of vital importance for the long-term outcome. Here we report that in an unselected, population-based cohort of breast cancer patients, the efficacy of the Swedish BRCA testing criteria was 65%. However, the effectiveness, corresponding to real world circumstances, of these criteria was only 18%.

BRCA testing in Sweden has much been driven by clinical geneticists and with a family history perspective, and with an emphasis on the autonomy of the individual tested. The Swedish BRCA testing criteria have only been changed marginally over the past 15 years, and are currently more strict than in many other countries [28, 29]. There has long been debate how to refine testing criteria, in order to improve sensitivity and specificity. We agree with Finch et al. [6] that a 10% a priori probability of carrying a mutation to merit testing should now be considered outdated. Less strict criteria would allow for more mutation carriers to be identified. As an example, only 2 out of 20 mutation carriers in our cohort did not meet the current NCCN guidelines for genetic testing [28].

However, according to the results of our study, improving efficacy of BRCA testing criteria will not be enough, if the difference between efficacy and effectiveness is not also reduced. It should be pointed out that a person’s decision to pursue genetic testing is preference-sensitive. For a preference-sensitive decision, the goal is to make a “quality” decision rather than a “right” decision [30]. For example, two out of six patients referred for genetic counseling in our study did not attend genetic counselling; possibly they truly did not want to know about any mutation carriership. In any respect, we consider an effectiveness of 18% to be unacceptably low.

Even if the testing criteria in Sweden are strict, we do not believe that the difference between efficacy and effectiveness is more pronounced in Sweden than in other countries. In Sweden, all treating physicians are expected to adhere to only one set of criteria that are easy to understand. Furthermore, medical insurance coverage issues and access to genetic counseling and testing do not represent a barrier in Sweden, which could be the case in countries without comprehensive and universal health care.

Bias can never be fully accounted for in studies on genetic testing, but we believe that our study design and population-based sample makes our study less subject to bias than previous studies on effectiveness of criteria for genetic counseling/testing. On the other hand, the cohorts in some of the other studies are larger than our cohort. Some previous studies are based on questionnaires sent home to breast cancer patients, resulting in selection bias and possibly also recall bias [10, 13, 17, 21]. Most previous studies have only included patients under a certain age [9, 10, 13–17, 19, 21], which could overestimate effectiveness of testing criteria, since younger patients are more likely to be referred and tested than older patients [14, 17, 21]. Within a high-risk group of patients, family history criteria (identifying older carriers) are more often overlooked than age criteria [17].

Moreover, the recruitment setting profoundly affects the uptake rates of genetic testing [30]. The uptake rates reported in study cohorts from academic centers [14, 18–20], medical insurance companies [12, 15, 16], and quality-focused practices [22] might therefore not be representative of the true figure in a country.

Taken together, bias in previous studies is likely in the direction of overestimating effectiveness of testing criteria. We expect that effectiveness rates of testing criteria will decrease the nearer one approaches an average of real world circumstances. When universal testing of all breast cancer patients is being discussed as an alternative to any selection criteria at all, this must be taken into account, favoring universal testing.

Van den Broek et al. [9] retrospectively analyzed a large cohort of Dutch breast cancer patients diagnosed below the age of 50 in 1974–2002 for a panel of *BRCA1* and *BRCA2* mutations. Their results indicated that the sensitivity of the Dutch referral criteria, corresponding to efficacy, was 75.5% in the young age group of patients studied. Among patients diagnosed after 1994, twenty-six out of 39 *BRCA1* or *BRCA2* mutation carriers identified in their research setting had been tested by April 2012, and were thus already clinically tested. However, the high proportion of clinically tested patients does not correspond to the definition of effectiveness. Out of 26 clinically tested mutation carriers in the cohort of van den Broek et al. [9] 6 were referred prior to breast cancer diagnosis and 10 were referred >1 years after breast cancer diagnosis. In their paper, it is not stated how many of these mutation carriers that were actually tested as probands, and how many of them that underwent predictive testing at a later point of time, as a result of a mutation identified in a relative following new incident cases of cancer in the family. For comparison, in our study, 9 out of 20 mutation carriers had been clinically tested; in 3 of them, the mutation was already known in the patient or in her close family at time of diagnosis; 3 were tested as probands; the remaining 3 patients were not referred as probands, but underwent predictive testing at a later point of time. This illustrates the need of detailed information and unbiased samples to assess effectiveness.

The sample size is a limitation of our study, adding uncertainty to the point estimates. Also, it should be noted that the patients in our cohort were diagnosed with breast cancer in 2007–2009. Others have found that both referral rates and uptake of genetic testing has increased over the last decade, and our results can therefore not automatically be generalized to present circumstances [14, 15, 18, 19]. Furthermore, due to differences in selection criteria, funding, health care systems, public and professional awareness of hereditary breast cancer etc., effectiveness should ideally be studied in every country.

One patient in our present study population is a carrier of the stop-gain mutation *BRCA2* c.6901G > T. In this paper, she is considered a mutation carrier. Although we believe the mutation to be potentially pathogenic, stop-gain mutations in exon 12 could at this point not be classified as definitely pathogenic [27]. Therefore, we also did a separate analysis excluding this patient. In that analysis, efficacy was 68% and effectiveness was 19%.

In conclusion, we found that effectiveness of BRCA testing criteria was much lower than the efficacy. Our results indicate the room for improvement is larger regarding effectiveness than regarding efficacy, and that attention must be paid to the whole BRCA testing procedure, not only the definition of testing criteria. Detailed and representative studies on effectiveness of BRCA testing criteria, and ways to improve effectiveness, are needed.

## Electronic supplementary material

Below is the link to the electronic supplementary material.

Supplementary material 1 (DOCX 20 kb)
