# Supplementary material for: Efficacy versus effectiveness of clinical genetic testing criteria for BRCA1 and BRCA2 hereditary mutations in incident breast cancer
Source: Fam Cancer. 2017 Jan 24;16(2):187–93. doi: 10.1007/s10689-016-9953-x (PMC5357494; doi:10.1007/s10689-016-9953-x)
Supplement: Supplementary file 1 — Supplementary material 1 (DOCX 20 kb) [file 10689_2016_9953_MOESM1_ESM.docx]

| **Table S1. Patient selection** |  |  |  |  |  |  |  |
| --- | --- | --- | --- | --- | --- | --- | --- |
|  |  |  |  |  |  |  |  |
| **Clinical parameter** | **ABiM study patients** | | **Study cohort** | | **Patients not included** | |  |
|  | **n** | **%** | **n** | **%** | **n** | **%** | ***P*-value** |
| **Total number of patients** | 538 | 100 | 273 | 100 | 265 | 100 |  |
| **Age at diagnosis** |  |  |  |  |  |  |  |
| Median | 63 |  | 62 |  | 63 |  | 0.29 |
| Range | 29–92 |  | 29–92 |  | 30–92 |  |  |
| **Tumor size (mm)** |  |  |  |  |  |  |  |
| ≤ 10 | 79 | 15,2 | 14 | 5,1 | 65 | 26,3 | <0.001 |
| 11–20 | 218 | 42,0 | 131 | 48,2 | 87 | 35,2 |  |
| > 20 | 222 | 42,8 | 127 | 46,7 | 95 | 38,5 |  |
| Unknown | 19 |  | 1 |  | 18 |  |  |
| **Lymph node status** |  |  |  |  |  |  |  |
| N0 | 315 | 64,0 | 155 | 59,2 | 160 | 69,6 | 0.02 |
| N1–N3 | 177 | 36,0 | 107 | 40,8 | 70 | 30,4 |  |
| Unknown | 46 |  | 11 |  | 35 |  |  |
| **Nottingham grade** |  |  |  |  |  |  |  |
| 1 | 100 | 19,2 | 31 | 11,4 | 69 | 27,8 | <0.001 |
| 2 | 212 | 40,8 | 96 | 35,3 | 116 | 46,8 |  |
| 3 | 208 | 40,0 | 145 | 53,3 | 63 | 25,4 |  |
| Unknown | 18 |  | 1 |  | 17 |  |  |
| **ER** |  |  |  |  |  |  |  |
| Negative | 83 | 16,0 | 50 | 18,3 | 33 | 13,4 | 0.15 |
| Positive | 436 | 84,0 | 223 | 81,7 | 213 | 86,6 |  |
| Unknown | 19 |  | 0 |  | 19 |  |  |
| **PR** |  |  |  |  |  |  |  |
| Negative | 122 | 23,5 | 70 | 25,6 | 52 | 21,1 | 0.25 |
| Positive | 397 | 76,5 | 203 | 74,4 | 194 | 78,9 |  |
| Unknown | 19 |  | 0 |  | 19 |  |  |
| **HER2** |  |  |  |  |  |  |  |
| Negative | 445 | 86,4 | 228 | 83,5 | 217 | 89,7 | 0.053 |
| Positive | 70 | 13,6 | 45 | 16,5 | 25 | 10,3 |  |
| Unknown | 23 |  | 0 |  | 23 |  |  |
| **Ki-67** |  |  |  |  |  |  |  |
| ≤ 20 | 224 | 60,7 | 142 | 54,0 | 82 | 77,4 | <0.001 |
| > 20 | 145 | 39,3 | 121 | 46,0 | 24 | 22,6 |  |
| Unknown | 169 |  | 10 |  | 159 |  |  |
| **St. Gallen subtype** |  |  |  |  |  |  |  |
| Basal | 59 | 15,1 | 38 | 14,3 | 21 | 16,8 | 0.002 |
| Luminal A | 214 | 54,9 | 136 | 51,3 | 78 | 62,4 |  |
| Luminal B HER2– | 75 | 19,2 | 56 | 21,1 | 19 | 15,2 |  |
| Luminal B HER2+ | 27 | 6,9 | 26 | 9,8 | 1 | 0,8 |  |
| Non-luminal HER2+ | 15 | 3,8 | 9 | 3,4 | 6 | 4,8 |  |
| Unknown | 148 |  | 8 |  | 140 |  |  |
| **Present or past CBC at time of diagnosis** |  |  |  |  |  |  |  |
| No | 516 | 95,9 | 261 | 95,6 | 255 | 96,2 | 0.83 |
| Yes | 22 | 4,1 | 12 | 4,4 | 10 | 3,8 |  |
| **Neoadjuvant chemotherapy** |  |  |  |  |  |  |  |
| No | 510 | 99 | 271 | 100 | 239 | 98,0 | 0.02 |
| Yes | 5 | 1 | 0 | 0 | 5 | 2,0 |  |
| Unknown | 23 |  | 2 |  | 21 |  |  |
| **Adjuvant chemotherapy** |  |  |  |  |  |  |  |
| No | 308 | 68 | 147 | 60,5 | 161 | 76,7 | <0.001 |
| Yes | 145 | 32 | 96 | 39,5 | 49 | 23,3 |  |
| Unknown | 85 |  | 30 |  | 55 |  |  |
| **Adjuvant endocrine therapy** |  |  |  |  |  |  |  |
| No | 138 | 29,9 | 63 | 25,2 | 75 | 35,4 | 0.02 |
| Yes | 324 | 70,1 | 187 | 74,8 | 137 | 64,6 |  |
| Unknown | 76 |  | 23 |  | 53 |  |  |
